# LiEIF and its recombinant polypeptides enhance the maturation of mouse dendritic cells and the production of the protective IL-12 cytokine

**Published:** 2011-01-10

**Authors:** O Koutsoni, M Barhoumi, I Guizani, E Dotsika

**Affiliations:** 1 Laboratory of Cellular Immunology, Department of Microbiology, Hellenic Pasteur Institute, Athens 11521, Greece; 2 Laboratoire d’Epidémiologie et d’Ecologie Parasitaire, Institut Pasteur de Tunis, 1002 Tunis-Belvedère, Tunisia

## 

Dendritic cells (DCs) maturation is associated with upregulation of costimulatory molecules (CD80, CD86, CD40) and secretion of cytokines including IL-12 which is important for generation of effective T cells. Proteins of *Leishmania* parasite that stimulate the production of IL-12 could be of significant interest either as immunotherapeutic components for leishmaniasis or as adjuvants. LeIF (*Leishmania* eukaryotic initiation factor) belongs to this group of proteins since it induces the production of IL-12, IL-10 and TNF-α by human monocytes of healthy volunteers. In particular, the induction of cytokines appears to be located in the N-terminal part (1-226) of the protein. In the present study we evaluated the ability of the recombinant protein *L. infantum* eIF (LieIF) and its constructs to induce *in vitro* the maturation of myeloid DCs (mDCs) and the production of cytokines supporting the polarization of Th1 type immune response. For this purpose, we used five synthetic peptides (16-18 aa) belonging in the N-terminal region, eight overlapped recombinant polypeptides covering the full protein sequence and the full-length protein. Enriched mDCs were obtained by *ex vivo* expansion of bone marrow cells, from BALB/c mice, cultured with the hematopoietic factor GM-CSF. The incubation of mDCs with the recombinant polypeptides, led to their maturation since it was observed a significant augmentation of the percentage of mDCs that express the molecules CD40, CD80 and CD86. On the contrary, the synthetic peptides did not enhance CD40 and CD86 expression and drove only to significant augmentation of CD80. In addition, we evaluated the ability of LieIF and its polypeptides to induce the production of IL-12, IL-10 and the expression of iNOS by mouse mDCs. We determined an augmentation of the percentage of mDCs that produce IL-12 upon their incubation with LieIF as well as with all the recombinant polypeptides and with three synthetic peptides whereas negligible amount of IL-10 was obtained. Augmentation of iNOS was found after incubation with the entire molecule of LieIF and with the recombinant polypeptides. In conclusion, LieIF and some of its recombinant polypeptides seem to have immunomodulatory properties demonstrating their potential use as therapeutic and prophylactic vaccine antigens.

